# Pharmacologia; Being an Extended Enquiry into the Operation of Medicinal Bodies, upon Which Are Founded the Theory and Art of Prescribing

**Published:** 1843-07-01

**Authors:** 


PHARMACOLOGIA; BEING AN EXTENDED ENQUIRY INTO THE OPERATION OF MEDICINAL BODIES, UPON WHICH ARE FOUNDED THE THEORY AND ART OF PRESCRIBING. By *J. A. Paris*, M.D. Edition Ninth. London: Highley, 1843.

IF the number of editions through which a book has passed, be a fair standard whereby to judge of its merits (and certainly a strong plurality, and high desert generally go hand in hand), the book now under consideration comes before the world with powerful recommendations. We shall present our readers with an analysis of the practical parts of it, interspersing our own remarks as we go along, Before, however, we enter on the more immediate subject of which the work treats, we cannot forbear expressing our decided and cordial concurrence in the truth and justice of certain remarks made by the author in the preface to his work. He states, and we think with much propriety, that although the medical public were already in possession of several pharmaceutical compendiums drawn up for the purpose of directing the practice of the junior, and of relieving the occasional embarrassments of the more experienced practitioner, and had books on materia medica, pharmaceutic chemistry, &c. sufficiently descriptive of the natural history, sensible qualities, chemical constitution and medicinal virtues of the several articles used in medicine, as also explaining the various pharmaceutical operations by which such bodies might be rendered available as remedies, still there was something yet wanted; no directions were given to teach the student, when the medicines were put into his hands, how he should mix, combine, and order their application in the form of extemporaneous prescription. It is well known to every experienced practitioner that, amidst all the perplexities which beset the young practitioner, there is none more embarrassing than that of adapting a prescription to all the circumstances of a particular case with therapeutical propriety and chemical accuracy. For the want of such guidance he is necessarily abandoned, on commencing his career of practice, to the alternative of two great evils—a servile routine on the one hand, and a lawless empiricism on the other. In the truth and value of these observations we repeat we entirely concur. We have only to regret that our author has not, in these remarks, pointed out the sources whence the student should derive, in the first instance, this knowledge which was to enable him to prescribe with therapeutical propriety and chemical accuracy; for surely he does not mean to say that his work was to fulfil these very important and essential ends; the former, viz. the prescribing with therapeutical propriety, if we understand the Doctor aright in his use of the terms, can only be obtained from an acquaintance with pathology, that is, in plain English, with the nature, symptoms, signs and seats of disease, as well as with that very particular and important branch of the science, viz. general therapeutics, namely, the various indications of treatment, and the most judicious methods of fulfilling them; such knowledge, combined with a thorough acquaintance with the physiological and therapeutical action of medicines will secure him sufficiently against the danger of therapeutical impropriety; whilst a knowledge of chemistry in general, as well as of the chemical habitudes and relations of the medicines employed, will keep the practitioner quite clear of the quicksands of chemical inaccuracy. These remarks we have been induced to make from our apprehending that the inexperienced student might be led to suppose that Dr. Paris intended that the study of his work was to supersede the necessity of perusing those works expressly devoted to chemistry, materia medica, and pathology. The author never meant such a thing. The object of his book is to teach the student the best method of applying the knowledge derived from the study of the above sciences, when he is called on to prescribe for the cure or alleviation of disease—and justice obliges us to say that his book will be found to effect this object most completely and satisfactorily. The author apologizes for omitting in the present edition that portion of his work which constituted the materia medica, referring the student to the excellent works of Christison and Pereira for information on that subject. In this we think Dr. Paris acted wisely.

In lieu of this part he offers the compensation of a much more extended view of that province, "which," he says, "I must continue to regard as peculiarly my own, for no author of the best repute has hitherto invaded it—the philosophy of medicinal combination." We have been so long accustomed to associate in our minds great modesty and great merit, that we very much regretted to meet such an assertion coming from Dr. Paris.

We fancy we hear some surly critic ill-naturedly exclaim "*exceptio probat regulam,*" in which exclamation we however do not join. Certainly Dr. Paris must recollect that Gaubius led the way in one of those departments which constitute the subject of his (Dr. P.'s) book in his celebrated work, entitled *Ars concinnandi formulas,* from which, by the way, modern medical writers are so fond of borrowing without the least acknowledgement; whilst the department treating of the classification, and modus operandi of medicinal substances had been anticipated by the late Dr. Murray. No doubt Dr. Paris has availed himself in the most dexterous, we mean of course the most judicious, manner, of the vast improvements which have been since introduced into the various branches of medical science; but he is in error when he says that the province of pharmacology is peculiarly his own; Gaubius and Murray invaded the province and conquered it; Dr. Paris took peaceable possession of it, and has made vast improvements in it—in a legal sense, however, we must admit that it is his own, possession being, as the saying is, nine points of the law.

Part I. which contains merely the Revolutionary History of the Materia Medica, we shall pass over, as not admitting of analysis, and shall proceed at once to—

Part II. In which are considered the Operations of Medicinal Substances, and the Classifications founded on them.

"Medicines are defined to be those bodies which by due administration are capable of producing changes in the condition of the living system, whereby its morbid actions may be removed or controlled." That *medi-*

*cines are for the most part but relative agents, producing their effects in reference only to the state of the living frame*, is a truth which our author is very anxious to impress on the mind of the young practitioner. He concurs with Sir Gilbert Blane in believing that the virtues of medicines cannot be fairly essayed, nor beneficially ascertained, by trying their effects on sound subjects, because that particular morbid condition does not exist which they may be exclusively calculated to remove. We must say we do not go the entire length with our author; we are inclined to think that the therapeutical effects of medicines (their virtues) should be deducible from their primary or physiological effects, at least there should be some connexion between them.

*The particular Organs, Fluids, and Tissues of the Body may be acted on through Four distinct Channels, or Modes of Communication.*

I. BY THE ACTUAL CONTACT OF THE APPROPRIATE REMEDY.

A. Conveyed through the medium of the circulation to distant parts, *without decomposition.*

Internally.

*a. Through the lacteal vessels.*
*b. Through the branches of the venæ portarum.*
*c. Through the capillaries.*
*d. Through the absorbents of the alimentary canal.*
*e. Through the absorbents of the bronchial vessels.*

Externally.

*f. Through the divided blood-vessels.*
*g. Through the lymphatics.*

B. Conveyed by absorption, *with decomposition*; by which one or more of its constituents are developed, and carried into the circulation.

*a. Through the same channels as in the preceding case.*

II. BY AN IMPULSE CONVEYED THROUGH THE INSTRUMENTALITY OF NERVES.

*a. Through the sympathy of their peripheral extremities.*
*b. Through the intervention of the nervous centres, and their reflex action.*

III. BY THE SYMPATHETIC CONTROL EXERTED BY THE STOMACH AND ALIMENTARY CANAL ON DISTANT PARTS.

IV. BY THE OPERATION OF CONTIGUOUS SYMPATHY, OR BY THAT WHICH IS EXCITED BY THE MERE PROXIMITY OF PARTS.

On each of these several divisions the author makes several remarks.

1. A. *On Remedial Agents conveyed by Absorption* WITHOUT DECOMPOSITION.

"That certain bodies are capable of evading the assimilating functions, and of entering unchanged into the circulation is a fact, capable of physiological, chemical, and therapeutical demonstration; thus, the physiologist has proved that a substance introduced into a closed cavity may disappear,—the chemist has traced it into the blood, and detected its presence in the secretions, or tissues of the body, while the physician has recognised its specific effects upon the organs with which it has come into contact, or through which it has passed. Among the substances which so pass, are the *carbonate, chlorate, nitrate,* and *sulpho-cyanite of potass,* and the *bi-borate of soda*—these pass unchanged through the blood, and are excreted by the kidneys. *Ferro-cyanuret of potass* (prussiate of potass) has been detected in the urine by Westrumb, from two to ten minutes after it had been swallowed, as well as by several other physiologists after different intervals.

" I have myself made many experiments upon this subject, and have verified to my entire satisfaction several of those of Christison, Coindet, Tiedemann, and others."

On the subject of the absorption of substances from the surface of the body, three distinct propositions are included: 1*st. With respect to the absorbing powers of the skin from simple contact:* 2*d. From friction.* 3*d. From the removal of the cuticle.* On these several questions, we beg leave to refer to the work itself.

A most interesting question has arisen in modern times, as to the mechanism of absorption. It is now very generally believed that there are no vessels terminating by open extremities, as Haller and others imagined; and even Bichat's idea of exhalant vessels being the open side-branches of the capillaries has been disproved. The opinion now prevails that all animal textures are permeable to fluids, by virtue of their pores, and that absorption is effected by what Majendie calls *Imbibition;* the phenomena of which, under the terms of *Exosmose* and *Endosmose,* have been fully investigated by M. Dutrochet.

" The medicinal substance, then, having, through one or more of the channels above stated, found its way into the general current of the circulation, the next enquiry is, as to what becomes of it. Here, then, arises another question of singular interest, and of great practical importance, as being one which will not only influence our theoretical views, but which must give a scientific direction to our practice. It is evident that the substance thus introduced must either be subsequently ejected from the body, through the medium of some of its secretions or excretions—or become united to some of its textures—or combined with, or wholly decomposed by, the vital action of the blood or its vessels."

" That certain remedies should act upon particular organs," says our author, " and leave others wholly uninfluenced, is a fact which appears to me to be far less mysterious than some physiologists have supposed. The substance in question necessarily pervades the whole organisation, through the medium of the circulating blood; but it will only affect such organs as possess *a peculiar susceptibility of its action.*"

The author should have seen that the " peculiar susceptibility" extricated him from no difficulty, save that of a *petitio principii,* or at least a something exceedingly like it.

Our author next considers the subject of—

1. B. *Remedial Agents conveyed into the System by Absorption,* WITH DECOMPOSITION, *by which one or more of the constituents are developed.*

In this category are included the greater number of substances that act by being absorbed; as there are comparatively few that do not undergo some change during their transit and final exit from the body. It was a maxim of Cullen's, that with respect to vegetables, and also some animal substances, "it is often a certain portion of them only that can be subjected to our digestive organs, while the medicinal part of the same is hardly affected, and therefore it may be alleged that their operation on the interior parts is not prevented by the powers of digestion." Our author lays claim to the merit of having been the first who attempted to give to this vague proposition a more definite form, or to examine the laws by which such decompositions may be governed. Since his having done so, writers on the Materia Medica have accepted the theory, and it now very generally enters into all speculations regarding the operation of medicinal bodies.

Our author adopts the classification of Dr. Murray, with a few additions of his own.

His classification is as follows;—A. GENERAL STIMULANTS. B. CONTRA-STIMULANTS. C. LOCAL OR SPECIAL STIMULANTS. D. CHEMICAL REMEDIES. E. MECHANICAL REMEDIES. F. ALTERATIVES. Under each of these six heads he arranges several classes. The first of these heads he sub-divides into *a*. Diffusible, and *b*. Permanent. To the Diffusible belong Exhilarants, Narcotics, and Anti-spasmodics, and to the Permanent, Stimulants, Tonics, and Astringents. The physiological correctness of this arrangement has been frequently disputed; it has been found, however, extremely difficult, nay even impossible, to substitute one wholly free from objection. We have often thought, that, if the primary effects of medicinal substances could be made the criterion of classification, much confusion might be avoided; we are well aware, however, that a serious objection could be made here also, as the primary effect itself is variable, depending, as it frequently does, on the quantity administered. The truth of the maxim, that medicines are mere relative agents, appears no where so striking as in the case now before us; let the system be languid and oppressed from intestinal accumulations, the operation of a purgative will, by unloading the bowels, relieve the state of languor and oppression, and so exhibit all the effects of a stimulant; let the brain or the lungs be congested, venesection will, by removing this state, arouse the powers of the system, and so act the part of a stimulant.

"The two most essential processes of animal life," says our author, "are nutrition and excretion, and these are exclusively performed by capillary vessels; suppose the balance of their circulation to be disturbed; mercury, by restoring and equalizing it, might, in certain cases, prove a true stimulant." In this small compass there is some physiology which may admit of question. In the first place, we are told that "the two most essential processes of animal life are nutrition and excretion"—it is usually taught that nutrition and excretion are by no means processes of *animal* life, but of *vegetative* or organic life—letting that, however, pass, what means "the balance of their circulation being disturbed"? —what precise idea is to be annexed to these words? what can, properly speaking, *unequalise* the circulation? we know that the circulation may become quicker or slower, stronger or weaker, or more or less irregular, in a part, but it cannot be *unequal*; the blood must be sent *equally* to every part of the body, passing as it does from the heart through a single canal—the aorta.

Again, we read, "the nervous system may be in a condition that repels sleep; a judicious and well-directed narcotic, in as much as it affords the means of indirectly *giving power* to the body, may be correctly considered as a *stimulant.*" The distinction is not clear between stimulants and tonics in this place. Stimulants give increased action; tonics give power; but, according to the author's own words, "action is not power." We fully agree with the author, when, he says, "we must at once perceive how imperfect must be every classification that does not acknowledge pathology as its foundation." This is sound, orthodox doctrine.

"From the too common error of mistaking action for power, and of regarding that which is, in fact, only borrowed, for an actual increase of capital, the practitioner has often concluded, that debility and stimulants (I use the terms according to their popular acceptation) constantly stand towards each other in the relations of disease and remedy; the same false reasoning will also lead him to conclude that a debilitated patient can never bear depletion, forgetting that the weakness may be the effect of disease, the removal of which can alone restore strength. The practical applications of these views are as numerous as they are important, and we shall have frequent occasion to refer to them."

Where the debility is *real*, where it is the effect of previously impoverished diet or of long-protracted disease, accompanied by great inanition of the capillaries, then we would have a poor opinion of that man's practice who would have recourse to depletion; but when the debility is only apparent, when it is *oppression* and not debility, that really exists, such oppression being occasioned, as so frequently happens in fevers, by the congested and adynamic state of the lungs, in which the blood is but imperfectly arterialized, then the brain, being thus supplied with such deteriorated blood, becomes oppressed, as does the entire nervous system with it—here to employ stimulation would but tend to overload still further the already congested capillaries of the brain, and so to oppress the nervous system that the powers of life must of necessity ultimately decline. Here the obvious indication is to employ depletion, by which means the lungs, brain, and other organs which may be in a state of congestion become relieved of the load which oppresses them. The lungs, thus relieved, become better able to arterialize the blood; and this improved blood becomes better adapted for the restoration of the functions of the nervous system. The great point in such cases is carefully to distinguish real from apparent debility.

We now come to the interesting subject of *Narcotics,* which our author defines to be "substances which, in moderate doses, occasion a temporary increase of the actions of the nervous and vascular systems, but which is followed by a greater depression of the vital powers than is commensurate with the degree of previous excitement, and which is generally followed by sleep." When large doses are taken, the symptoms of diminished sense and motion follow so rapidly, that the previous stage of increased action is very obscure, or not at all perceptible. This two-fold effect of narcotics now seems to admit of explanation from the fact that they, or at least the principal one, *opium,* contain both stimulant and narcotic properties combined, the former depending on the presence of *narcotine,* the latter on that of *morphia.* The circumstance that the narcotic principle is generally combined with either a stimulant or a sedative renders it peculiarly necessary to distinguish it from both the one and the other of these; this fact it is which has occasioned the contradictory and unsatisfactory reports of the value of the different narcotic remedies. It is rather surprising that our author makes no allusion whatever to the co-existence of these different principles, the more so as it is on a knowledge of it that the judicious employment of them depends in the treatment of disease. Thus opium, which contains a stimulating principle combined with the narcotic, will not suit those cases of disease in which hyosciamus, which contains the narcotic combined with a sedative, would be applicable. If we want to administer a narcotic to a person who exhibits the symptoms of pyrexia, as hot skin, thirst, delirium, &c., we should prefer hyosciamus or some sedative narcotic to opium, by which no doubt the patient will be put to sleep, but sleep from which he is likely to awaken feverish and unrefreshed, whilst a sedative narcotic would have cooled and refreshed him. There is, however, something in the action of opium, which we must admit stands very much in need of elucidation.

We next come to the consideration of CONTRA-STIMULANTS, or SEDATIVES. It has been the custom to identify these and narcotics, and to place them in the one category; however, as our author well observes, whoever has carefully observed and compared the effects of these agents must withhold his assent to such an alliance. "A *sedative*, in whatever dose it may be given, is never followed by the slightest indication of excitement: it directly and *primarily* depresses the powers of life, whereas a narcotic in small doses never fails to increase the vital force." In fact, a sedative produces its effects by repressing the nervous influence, and thereby diminishing the action of the heart and other organs. Some will have it, however, that there is no such thing as a direct, primary sedative; but that the sedative effect was only the secondary result of exhaustion from stimulus. To this conclusion they are led from the state of stupor which is observed to supervene on intoxication after the use of fermented liquor; as also from opium, which is observed to be stimulant in small quantities, before enough of it is taken to produce stupor. A state of exhaustion being thus observed to follow intoxication or any other abuse of stimulus, it has been taken as a matter of course that every sedative effect must be secondary, and the consequence of previous stimulation.

Our author's observations on the physiological effects of sedatives, as well as of their application to the treatment of disease, are not so copious as he might have made them. Substances of this class, when taken in a large dose, are observed to produce great anxiety and despondency of mind, and to render the nervous system so depressed that it becomes incapable of directing the muscles—hence the giddiness and staggering so often observed after the use of cigars in excess: the retina itself is often so much affected, that the individual cannot see distinctly: we know the same effects may be produced by severe hæmorrhage. We know that coma may arise from either plethora or inanition; its treatment must evidently depend upon which of these two different and opposite states have occasioned it; if it arise from inanition, as it sometimes does in the case of children, depletion would be mischievous, and we must have recourse to stimulants; whereas, if it be occasioned by congestion, the opposite line of practice is indicated. A knowledge that opposite states may give rise to the same symptoms is of the utmost importance in the practice of medicine.

Sedatives, contra-distinguished from stimulants, diminish the injection of the brain, at the same time repressing nervous influence; whilst stimulants, by exciting the heart's action, and also increasing the discharge of nervous influence, increase the injection of the brain: hence when stupor or delirium comes on from sedatives, inanition is the proximate cause, whilst congestion is the proximate cause when stimulants produce such symptoms.

*Antispasmodics.*—These, our author defines to be, "substances which are supposed to possess the power of allaying the inordinate action of muscular structures." The necessity of establishing a distinct class of substances, as capable of *specifically* controlling spasmodic action, is, to say the least of it, very questionable.

Before we can determine how far we are justified in recognizing any class of bodies capable of controlling spasm, we must first know what spasm is. Spasm has been defined to be a temporary, irregular, and involuntary action of muscles, attended with more or less pain, and followed by exhaustion. "The general cause would appear to be morbid impressions upon the nervous system, of which the following are the principal exciting causes.

1. Irritation of the nervous centres.
2. A loss of balance between the nervous and sanguineous system.
3. Irritation in the primæ viæ.
4. Cold.
5. Excessive muscular re-action excited by over-extension.
6. A laborious effort to expel foreign matter."

Thus, then, it is to the cause of spasm, viz. the cause of nervous irritation, and not to the inordinate muscular action itself, that the physician is to direct his attention. Indications of a plethoric or inflammatory condition of the brain or spinal marrow are to be met by depletion; whilst symptoms indicating sanguineous inanition, as spasm may arise from those opposite states, is to be combated by the appropriate treatment. Narcotics, from their power of allaying irritation and pain, are found to be very efficacious in the treatment of spasm. Spasm, in fact, depends on a variety of causes, and in order to treat it judiciously, the cause must be first ascertained—purgation, venæsection, tonic treatment, stimulation, sedatives, narcotics, will, in their turns, serve to allay it, according to the causes on which it may depend.

The influence of the mind in encreasing or allaying spasm must not be left out of consideration in the treatment of this affection. There is nothing more likely to produce spasm, than an excessive degree of sedative action—thus excessive hæmorrhage, the action of lead, sour fruit, &c., are frequently followed by spasm. This circumstance will point out the indispensable necessity of investigating the cause, and absurdity of prescribing for mere symptoms—sedatives, narcotics, stimulants, &c., may evidently be indicated according to the cause.

*Tonics.*—We now come to *Tonics*, which the author defines to be "substances, whose continued administration gives strength and vigour to the body, without producing sudden excitement, or subsequent depression."

Of course this definition is a make-shift—it does not contain in it that great essential of a good definition, namely, adequacy—but, to say the truth, it is not so much the author as the nature of his subject we have to blame.

"When the vital movements have been accelerated by a stimulant beyond a certain point, the consequence will be a corresponding collapse, as already explained, and an interval must elapse before the exhaustion can be supplied, and the natural balance re-established through the agency of the *vital stimuli;* not so, however, with Tonics, for as they act slowly, and yet progressively, time is allowed for the full operation of the vital stimuli to supply an influx of power which shall, at least, equal the demand for it, and consequently no collapse can take place: but more than this is effected; for, since the reanimating or restorative functions will necessarily partake of the general excitement, they will be urged with increasing activity, and thus, as it were, by an ascending scale, will the energies of every part be gradually and permanently increased, and the general standard of strength raised. To ensure, however, this desirable result, we must be careful so to regulate the application of the vital stimuli, by a judicious system of *medical training,* as shall insure the full benefit of their revivifying influence. Such is the theory by which I propose to explain the operation of tonics, and the phenomena will be found to correspond with it; thus to the above conditions there must evidently be a limit; when the powers of the system have been urged to a certain amount, or as far as may be consistent with its well-being, the tonics assume the character of an excitant; the vital stimuli are no longer adequate to the demand made upon them, and indications of collapse present themselves."

This is rather metaphysical; and will, perhaps, scarcely suit the tastes of the plain matter-of-fact kind of folks now going. With this portion of the profession at the present day tonics are substances, which are neither stimulant nor sedative; they neither call forth actions, like the former, nor do they repress them, like the latter; their action is directed to the nervous system which they enable to generate the nervous influence, by which the entire system is invigorated. Tonics give strength, stimulants call it forth. There are occasions, no doubt, where stimulants produce tonic effects, viz. when, by stimulating the digestive organs, they enable them to perform their functions more vigorously; the new nourishment thus taken into the system, acts upon and strengthens the nerves as well as the other parts; but this does not prove that tonics are stimulants; we know there may be occasions where the employment of a purgative may be followed by tonic effects. We see sometimes the employment of a tonic followed by an increase in the strength and quickness of the pulse, with headache, &c. and this is set down as the effect of a stimulating property of the tonic; this, however, is the result of morbid sensibility of the stomach, occasioned by the tonic disagreeing with it, and not of any stimulant power inherent in the medicine. There is no class of medicines which go farther to prove the principle that medicines are relative agents, than tonics—venesection—emetics—purgatives—may prove tonic, according to the state of the system.

The therapeutical use of tonics, in the treatment of chronic inflammation, has not been adverted to by our author, though that is an extremely important part of their use in the practice of medicine; he has not touched upon the matter. The efficacy of tonics, however, in the treatment of chronic inflammations is undoubted. Bark, iron, mercury, nitrate of silver, are, all of them, found serviceable in the removal of chronic inflammations ; an effect which they seem to produce by their power of giving healthy tone to the capillary vessels of the affected part, when conveyed to them through the circulation—the efficacy of bark in the treatment of erysipelatous inflammation, when the acute stage has passed by is well known—the beneficial effects of mercury in effecting the resolution of chronic inflammations, is also universally recognised. Even in acute inflammation, when the acute stage has been subdued, it may happen that the powers of the system are so depressed, that the patient appears likely to sink: in such a case, the exhibition of tonics becomes of the utmost importance. Rheumatic inflammation in the fibrous and other tissues, the capillaries of which are very minute, are most effectually treated by mercury, iodine, arsenic, antimony, &c., introduced into the capillaries through the medium of the circulation. Analogy would incline us to consider that tonics are indebted for their efficacy in relieving inflammation to their astringency, that is, we consider them to produce an astringent effect on the relaxed capillaries of the affected part.

We now come to a class of medicines very near akin to *Tonics*, namely, *Astringents*, which our author defines to be " substances which, when applied to the living body, corrugate and condense its fibres; and at the same time exert a tonic influence through the medium of its living principle."

From this definition it would appear, that very little difference exists between astringents and tonics ; in fact, none whatever, if we admit that tonics produce their effects by constringing the capillary vessels.

Astringency is readily recognised by the organs of taste; the papillæ of the tongue become corrugated, and a sensation of roughness is communicated to the taste. Astringent substances are found in the mineral and vegetable kingdoms—thus, the mineral acids are found to be astringents, many of the metallic salts, also, especially those of iron, zinc, copper, and lead; some of the earths, also, combined with acids, as alum. The vegetable kingdom affords the greatest number of astringents; this principle is found to depend on a peculiar proximate principle called *Tannin*, or *Tannic Acid*, characterised by its forming with animal gelatine an insoluble compound, (*Leather*,) and with the salts of the peroxide of iron precipitates of a deep blue colour. With this acid there is another acid found almost universally associated, viz. *Gallic Acid*, which also precipitates the peroxide of iron, but not gelatine; this acid also possesses astringency. It has been recently ascertained that tannic acid is readily convertible into gallic acid by the absorption of oxygen. Some even maintain that, whatever gallic acid is obtained from galls is formed by the action of air on their tannic acid.

The *modus operandi* of vegetable astringents, has been explained by some on the principle of their action in tanning. May we not account for their effects on morbidly secreting surfaces in the same way as we explained the effects of tonics; viz. by their constringing the extreme vessels of the relaxed parts? Some of them may produce their effects by acting as sedatives, as *lead,* cold. Their power of restraining hæmorrhage, may be attributed to their influence on the stomach and intestines being extended by sympathy to the more remote capillaries. We know the effect of chewing a mouthful of coarse common salt in the mouth, in arresting hæmoptysis; this will render it easy to conceive the possibility of such a sympathy. The efficacy of astringents is much more palpable, when they are applied locally; thus we see that astringent lotions act like charms in that relaxed state of the capillaries occurring after inflammation of the mucous membrane of the eye; and, after a gorged state of the capillaries of the alimentary canal from any cause, there can be little doubt that a passive state of dilatation may continue, after the cause of irritation has been removed, and that, under such circumstances, the use of an astringent medicine may force the relaxed and dilated vessels to resume their natural diameter. In this way the benefit derived from the preparations of steel in certain chronic derangements of the alimentary canal has been accounted for.

When the use of astringents seems to be indicated, it becomes necessary carefully to consider the cause of the morbidly excessive discharge, as such discharge may depend on diametrically opposite states of the system—we know hæmorrhage may be accompanied by plethora, or by real actual debility accompanied by inanition, and that the astringent to be applied will be toto cælo different—whilst the lancet and other depletory measures are to be employed in the former case, they are totally contra-indicated in the latter. When the discharge depends on irritability, opium will prove the best astringent, or the salts of lead may be employed; in cases of diarrhœa, depending on the flow of acrid fluids into the intestines, by which their peristaltic action is increased, we may employ an astringent, properly so called, which will repress this discharge immediately and directly; or we may employ opium, which, though it may not arrest the discharge, may render the mucous surface insensible to its acridity; or we may, without either repressing the discharge or diminishing the sensibility of the mucous surface, obtund the acrimony of the discharge by giving an absorbent, such as magnesia.

It is in cases of hæmorrhages that the use of astringents is most important. *Internal* bleeding is now known, in almost every case, to depend on a relaxed state of the minute capillaries, which allows the blood to escape by a kind of exudation, no rupture of vessels whatever being present; and as this state may accompany congestion or relaxation, the hæmorrhage may be *active* or *passive*—in the former case astringents would be not only useless but injurious—hence the obvious necessity of carefully investigating the conditions of the system on which the hæmorrhage may depend.

[*To be concluded in our next.*]

